# Exploring Quinazoline Nitro-Derivatives as Potential Antichagasic Agents: Synthesis and In Vitro Evaluation

**DOI:** 10.3390/molecules29184501

**Published:** 2024-09-23

**Authors:** Citlali Vázquez, Audifás-Salvador Matus-Meza, Oswaldo Nuñez-Moreno, Brenda Michelle Barbosa-Sánchez, Victor Manuel Farías-Gutiérrez, Mariana Mendoza-Conde, Francisco Hernández-Luis, Emma Saavedra

**Affiliations:** 1 Department of Biochemistry, Instituto Nacional de Cardiología Ignacio Chávez, Mexico City 14080, Mexico; cita2812@gmail.com (C.V.);; 2 Department of Pharmacy, Faculty of Chemistry, Universidad Nacional Autónoma de México, Mexico City 04510, Mexico; audifas.mateza@gmail.com (A.-S.M.-M.); vicmafag@comunidad.unam.mx (V.M.F.-G.)

**Keywords:** antiprotozoal agents, benznidazol, Chagas disease, nifurtimox, quinazoline, quinazoline-2,4-6-triamine

## Abstract

*Trypanosoma cruzi* is a protozoan parasite that causes Chagas disease in humans. The current antichagasic drugs nifurtimox and benznidazole have inconveniences of toxicity; therefore, the search for alternative therapeutic strategies is necessary. The present study reports the synthesis, drug-likeness predictions, and in vitro anti-trypanosome activity of a series of 14 quinazoline 2,4,6-triamine derivatives. All compounds were tested against *T. cruzi* (epimastigotes and trypomastigotes) and in HFF1 human foreskin fibroblasts. The bioassays showed that compounds **2**–**4** containing nitrobenzoyl substituents at 6-position of the quinazoline 2,4,6-triamine nucleus were the most potent on its antiprotozoal activity. The effect was observed at 24 h and it was preserved for at least 5 days. Also, compounds **2**–**4** were not toxic to the human control cells, showing high selectivity index. The quinazoline nitro derivatives have potential use as antichagasic agents.

## 1. Introduction

Chagas disease (CD), or American trypanosomiasis, is a neglected tropical disease classified as a zoonosis caused by the hemoflagellate parasite, *Trypanosoma cruzi* [1]. This protozoan is transmitted to humans either by a blood-sucking reduviid triatomine as primary vector, congenital mother to child during delivery, transfusion of infected blood, organ transplantation from infected donors and laboratory accidents [2,3]. It is a widespread protozoan infection in Latin America where it ranks second place after malaria in prevalence and mortality due to vector-associated diseases. At least 6–8 million people are infected and 75 million at risk of exposure to infection. An estimated of 10,000 CD-related deaths are reported annually [3,4]. Recently, CD is becoming a reemerging health problem in non-endemic areas because of infected people emigrating to North America, Europe, and the Pacific region [5]. 

Clinically, human CD shows acute and chronic phases. The acute phase starts with acquisition of the parasite where blood testing shows high levels of parasitemia, lasts approximately 30 to 90 days, and is generally asymptomatic or with non-specific symptoms. During the chronic phase (several years or decades after infection), nearly 30% of the patients suffer from noticeable injuries in the heart and/or in the digestive and nervous systems; the rest of the patients remain in the indeterminate phase, where no or less severe symptoms may occur [2,3]. Since in the chronic stage the immune system of the host keeps replication of the parasite under control but not sufficient for elimination, blood testing shows low levels of parasitemia, which complicate the detection of latent infection in tissues [5,6]. 

There is no vaccine against CD and treatment of the infection relies on only two nitroheterocyclic drugs, benznidazole (Bnz): *N*-benzyl-2-(2-nitroimidazol-1-yl) acetamide, and nifurtimox (Nfx): (E)-*N*-(3-methyl-1,1-dioxo-1,4-thiazinan-4-yl)-1-(5-nitrofuran-2-yl) methanimine [7,8]. These drugs affect all developmental stages of *T. cruzi* (epimastigote of the insect vector, and trypomastigote and amastigote of the mammalian cell host). Bnz treatment is well tolerated in children; however, in adult patients, they cause systemic toxicity and adverse effects that include anorexia, nausea, vomiting, headache, central nervous system depression or maniacal symptoms, seizures, vertigo, paresthesia, peripheral polyneuropathies, and dermatitis; these cause high rates of treatment abandonment [4,8]. An additional element that complicates the pharmacological management of this disease is the different susceptibility of different parasite strains to these drugs [9,10]. Recently, ravuconazole (E1224) and fexinidazole have been tested in clinical trials, the first being effective only in combination with Bnz, whereas the second proved to be toxic and at low doses ineffective as an antiparasitic in monotherapy [11,12]. As a consequence, there is a need for safe and efficacious new drug treatment, especially for the chronic phase [13]. 

The quinazoline nucleus is a privileged scaffold in the field of Medicinal Chemistry and it is one of the most promising heterocyclic moieties, which due to its easy functionalization, has been explored through structural modifications at various positions. Additionally, in silico studies have revealed that quinazoline analogues exhibited good binding affinity towards various receptors involved in several diseases [14]. The privileged status of quinazoline scaffold has been widely exploited in the design of new bioactive derivatives with different pharmacological profiles like anticancer, anti-inflammatory, antimalarial, antidepressant, antidiabetic, antihypertensive, and antimicrobial [15].

As part of our program on the discovery of novel antiparasitic compounds, we have focused on the quinazoline-2,4,6-triamine (TAQ) core because it is a scaffold structure that possess hydrogen bond acceptor, hydrogen bond donor, and π–π stacking interactions with the aromatic ring of amino acid residues, which makes it easy to form better interactions with drug target proteins [16]. In previous works reported by our group, some TAQ derivatives have proved to have an antileishmanial effect [17], whereas other different TAQ derivatives were studied as inhibitors of *T. cruzi* dihydrofolate reductase with favorable results [18]. 

In this paper, we report compounds **1**–**14** (Table 1) designed to evaluate the effect of substitutions benzoyl or benzyl at the 6-position of TAQ. In particular, in compounds **1**–**4** this substitution gives rise to the formation of an amide; in compounds **5**–**14** [19], the substituent gives rise to the formation of a secondary amine. These differences confer different electronic environmental that would affect the lipophilicity and the activity as antiparasitic drugs. In order to describe the potential for good oral bioavailability and to evaluate the drug-likeness of the designed compounds, physicochemical properties were predicted in silico using SwissAdme platform. The compounds were tested against *Trypanosoma cruzi* epimastigotes and trypomastigotes and its toxicity was evaluated against human fibroblasts. Also, for the most potent and selective compounds as antitrypanocydals, their effects on the antioxidant metabolite levels in the parasite were evaluated. 

## 2. Results

### 2.1. Chemistry

The strategy envisaged in the present study involved the preparation of 14 quinazoline-2,4,6-triamine derivatives disclosed in Table 1; for 8 of them, their syntheses have been previously described [19]. In a first stage, in silico predictions of some of their physicochemical properties were made. In a second stage, the target compounds were synthesized, and finally, the biological evaluations were carried out.

### 2.2. Drug-Likeness Predictions

The capability of a drug to exhibit a pharmacological effect depends on several of its physicochemical properties associated with its pharmacokinetic profile [absorption, distribution, metabolism, and elimination (ADME)]. Nowadays, these predictions are studied in initial phases of drug development by in silico approaches to generate potential drug-likeness molecules. In the present study, the in silico computational studies of the target quinazoline derivatives were performed using the online server Swiss ADME prediction tools (http://www.swissadme.ch/index.php; accessed on 25 February 2023) [20]. The drug-likeness parameters water solubility (S), lipophilicity (ClogP), topological surface area (TPSA), Lipinsky rule, Veber rule, percentage of absorption (%ABS), and gastrointestinal absorption (GI-ab) were predicted (Table 1). It is worth mentioning that inappropriate values of physicochemical properties are the major cause of attrition of the most drugs in the clinic, making profiling for the ADME endpoint an important pursuit in early drug discovery.

The data presented in Table 1 indicate that all the proposed TAQ derivatives have acceptable drug-likeness profiles to be considered in the subsequent biological evaluations. Some of the considerations we can mention are that compounds **2**–**4** showed the highest aqueous solubility values (0.3 mg/mL) of the entire set of compounds; however, they presented one Veber rule violation (TPSA > 140 Å^2^), which results in their having a lower potential to permeate biological membranes (ABS < 60%, Low GI-ab). On the other hand, compounds **1** and **5**–**14** showed a moderate or low solubility profile but with a high capacity to move through biological membranes (ABS > 60%, High GI-ab). According to Lipinski’s rule, any molecule that presents 0 alerts in the following parameters can be considered for biological evaluation: hydrogen bond donors < 5, hydrogen bond acceptor < 10, molecular weight below 500, calculated log *p* < 5, given that it has a good perspective for not showing absorption problems in the experimental organisms [20].

Finally, the negative PAINS parameter indicates that none of the compounds would give false positive results in biological assays by interacting indiscriminately with many classes of cellular receptors [21].

### 2.3. TAQ-Derivative Synthesis

**Scheme 1:**
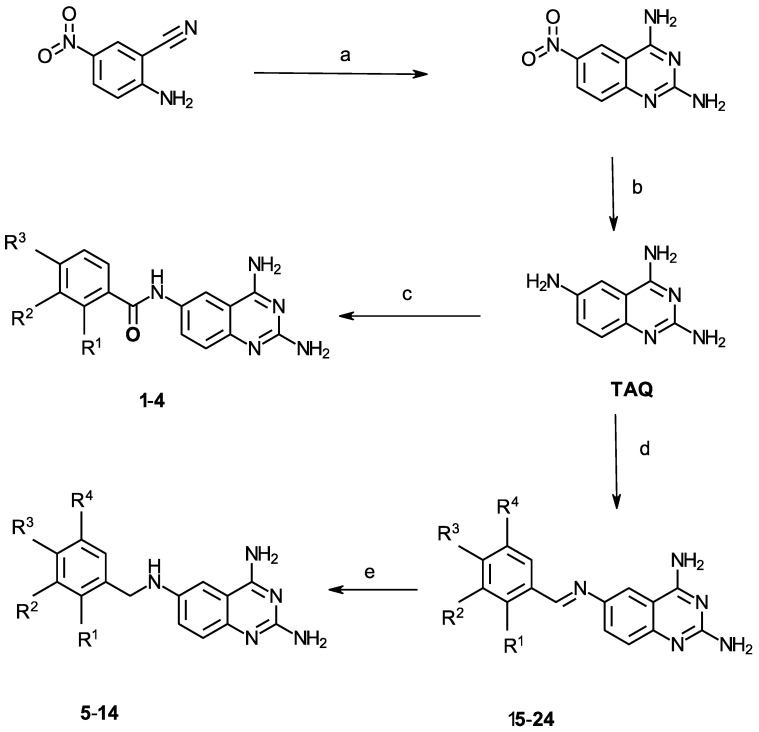
Reagents and conditions: (a) guanidine hydrochloride, NaOH, EtOH/PrOH, reflux; (b) H_2_, 10% Pd/C, MeOH, r.t.; (c) benzoyl chloride, DMF, triethylamine; (d) 1.2 eq. of aryl aldehyde, 10 drops DMF-DMA, 40 °C, 12 h; (e) MeOH, 60 °C, 1 h, then NaBH4 r.t., 1 h.

Quinazoline-2,4,6-triamine served as the starting material for the synthesis of compounds **1**–**14**. It was prepared by carrying out the cyclization of 2-amino-5-nitrobenzonitrile with guanidine carbonate to give 6-nitroquinazoline-2,4-diamine, followed by hydrogenation under a hydrogen atmosphere by using palladium carbon (Scheme 1). The starting material was subjected to methodologies previously described by our work group [18], with slight modifications in solvent and temperature. 

Compounds **1**–**4** were obtained by reacting TAQ with the corresponding acyl chloride using DMF as solvent at 25 °C. All products were obtained in solid form.

The imine intermediates (**15–24**) were obtained through a Schiff base reaction between the corresponding aldehydes 2 at 40 °C, with methanol as a solvent and N,N-dimethylformamide dimethyl acetal (DMF-DMA) as a dehydrating agent. These imines turned out to be very stable and were produced in yields from 60–80%. They were subjected to reductive amination by utilizing NaBH_4_ to give **5**–**14**. The syntheses of compounds **5**–**10**, **12**, and **13** have been previously described [19].

The structures of the target compounds were confirmed by ^1^H and ^13^C NMR and IR spectroscopy as well as HRMS spectrometry. 

### 2.4. In Vitro Effect of the Compounds on Parasite Forms and Mammalian Cells at Short Times

**Figure 1:**
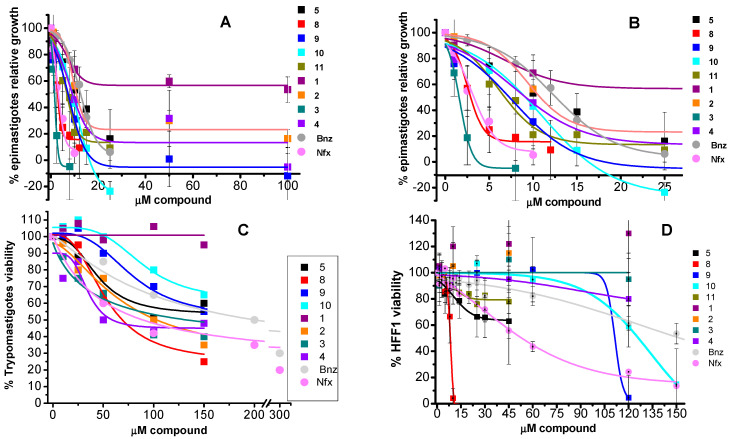
Effect of TAQ derivatives on parasites and cells. Effect of TAQ-derivative treatment on the growth of epimastigotes (**A**,**B**), viability of trypomastigotes (**C**), and viability of HFF1 fibroblasts (**D**). Panel B is a zoom from panel A at low drug concentrations for clarity. Treatments were 24 h for epimastigotes, 3 h for trypomastigotes and 24 h fibroblasts. The data plotted are the mean ± SD (n at least three independent cultures); when no S.D is shown, the data are the average of n = 2 independent cultures. In (**A**,**B**), the relative growth is the difference of the parasite density displayed after 24 h in the presence of the treatment minus the parasite density at time 0. 100% epimastigotes relative growth = 515,000 ± 58,000 parasites/mL; in (**C**) live trypomastigotes number = 235,000 ± 15,000 and in (**D**) HFF1 number of viable cells = 27,000 ± 6300 cell.

Compounds **1**–**14** were evaluated on the growth of epimastigotes cultured in vitro and viability of trypomastigotes (obtained from infected cells) of the virulent *T. cruzi* Queretaro strain [22] after 24 h and 3 h treatment, respectively (Figure 1). Compounds **2**–**5**, and **8**–**11** were effective in decreasing by 80% the growth of epimastigotes at doses lower than 20 µM (Figure 1A,B) and diminished the viability of trypomastigotes 30–70% at doses around 150 µM (Figure 1C). These inhibitions were higher or similar than those shown by Bnz and Nfx. In contrast, compounds **6**–**7** and **12**–**14** showed >30 µM or no effect at 100 µM (Supplementary Figure S1). 

The compounds were also evaluated on the viability of human foreskin fibroblasts (HFF1) as a model of human cells after 24 h treatment (Figure 1D). Bnz at concentrations > 50 µM and Nfx >15 µM started to show cytotoxicity. Remarkably, compounds **1**–**4** showed no cytotoxicity (and even an increased in proliferation was observed), whereas its base compound **1** (lack of nitro functional group in the benzoyl substituent) did not show either cytotoxicity or parasiticidal effects. Compounds **5** and **8**–**11** showed similar cytotoxicity as Nfx and higher than Bnz.

### 2.5. In Vitro Effect of the Compounds on Parasite Forms and Mammalian Cells after 5 Days

**Figure 2:**
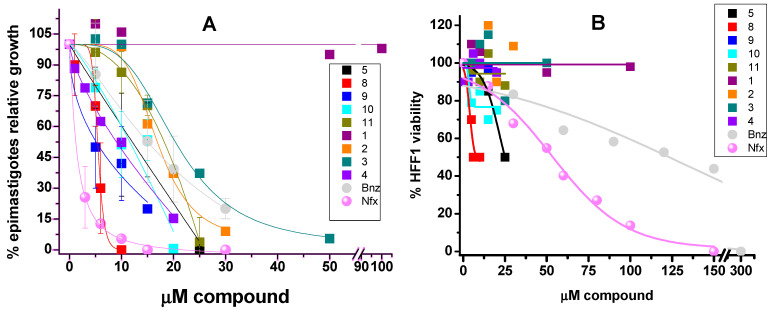
Effect of TAQ derivatives on the growth of epimastigotes (**A**) and viability of HFF1 fibroblasts (**B**) after 5 days. The data are the average of at least two independent cultures and of at least three with S.D.

To determine the long-term efficacy of the treatment with the TAQ derivatives, in another set of experiments, the treated epimastigotes were cultured and further monitored for 5 days to determine whether parasite growth recovery could be attained (Figure 2A). No regrowth of the parasites and no change in the order of potency among the compounds was observed. These suggested that damage was permanent and/or compounds were stable during treatment. Remarkably, compounds **1**–**4** showed no cytotoxicity up to 50 µM (Figure 2B) against HFF1 cells. In contrast, Bnz and Nfx showed high cytotoxicity, particularly Nfx. Compounds **5** and **8**–**11** showed higher or similar cytotoxicity than Nfx and Bnz. 

### 2.6. Inhibitory Concentrations

The inhibitory concentrations (effective dose 50: ED_50_ and lethal dose 50: LD_50_) on the relative growth for epimastigotes and cytotoxicity for trypomastigotes and HFF1 cells after treatment with the TAQ derivatives **1**–**14** is summarized in Table 2. 

Compounds of the series **2**–**4** characterized by the presence of a nitrobenzoyl group bound by an amido group to the quinazoline moiety showed good efficacy against both parasite forms, with no effect on HFF1 cells in short and long exposures; these resulted in higher selective index compared to Bnz and Nfx. Its base compound **1** with a benzoyl substituent without nitro groups did not showed an effect in parasites nor in cells.

In contrast to compound **1**, compound **5** with a benzyl ring substituent bound to the quinazoline moiety by an amine group was more toxic than the first. Compounds **6**–**7** with nitro groups at different positions in the benzyl ring of compound **5** lost parasiticidal effectiveness respect to their base compound **5** and compared to compounds **2** and **4**. 

Regarding halogenated TAQ derivatives, compound **8** was highly toxic for parasites and fibroblasts; **9**–**11** showed parasiticidal effect and good selectivity against epimastigotes, but lost effectiveness when tested against trypomastigotes. Other substitutions in the benzyl ring, i.e., compounds **12**–**14,** were not effective as an antiparasitic. 

### 2.7. Effect of TAQ Derivatives on Antioxidant Thiol Metabolites

**Figure 3:**
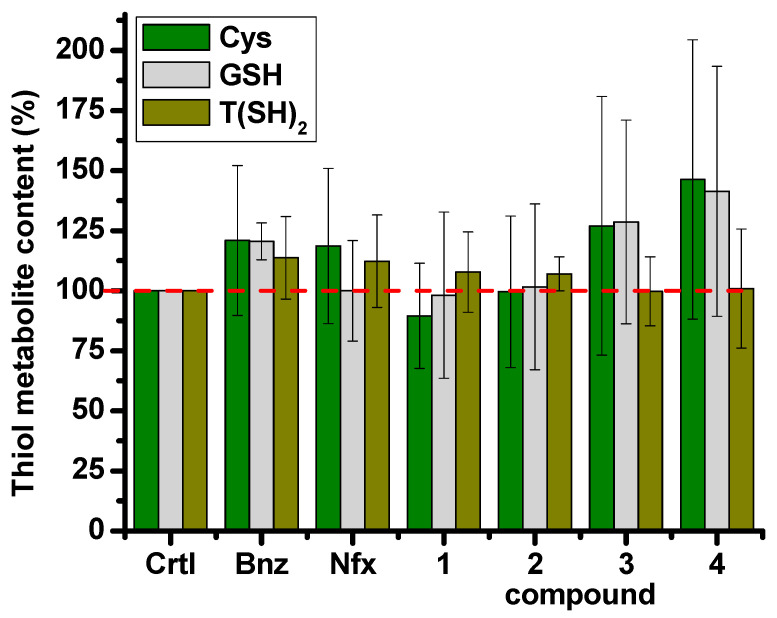
Effect of TAQ-nitroderivatives on the thiol metabolites of epimastigotes of *T. cruzi*. After 24 h of exposure to the ED_50_ and canonical antichagasics Bnz and Nfx, thiol metabolites were determined by HPLC. 100% correspond to Cys = 4 ± 1.4, GSH = 8 ± 2, T(SH)_2_ = 5.4 ± 2.4 nmol/mg cell protein. Data are the mean ± standard deviation of three independent parasite cultures.

To determine whether compounds with nitro groups 2–4 perturbed the antioxidant capacity of the parasites [23], the content of trypanothione (T(SH)_2_) and its precursors cysteine (Cys) and glutathione (GSH) were determined in epimastigotes exposed for 24 h at the ED_50_ of each compound (Figure 3). No significant changes were observed in T(SH)_2_, although a tendency to higher Cys and GSH were observed. Due to the high amount of biological sample required to determine thiol contents, the experiment could not be performed in trypomastigotes. 

## 3. Discussion

In vitro evaluation of the fourteen TAQ derivatives indicated that compounds **2**–**4** with a benzoyl with nitro groups bound to the quinazoline ring through an amide group have the highest parasiticidal effect. The compounds have an advantage over Bnz and Nfx due to their lower toxicity in a human cell model which resulted in a higher selective index. It is necessary to clarify that selective index was only calculated for epimastigotes versus HFF1 at 24 h and 5 days, in which the treatment regimes were similar. A selective index for trypomastigotes was not calculated, because for these parasite forms, the in vitro treatment cannot surpass 3 h due to the high transformation of trypomastigotes into amastigotes and mobility was used as parameter of viability, hence, the treatment regimes for trypomastigotes is quite different to that of HFF1 cells. However, the results with epimastigotes at 5 days can be interesting because in vitro culture epimastigotes have a proportion of intermediate forms that has been shown to be infective [24]. Notwithstanding, based on the results, compounds **2**–**4** can be proposed as potential antichagasic agents which deserve further studies. 

The mode of action of these compounds remains to be elucidated. Different TAQ derivatives were designed by docking analysis against dihydrofolate reductase and pteridine reductase enzymes from *T. cruzi* showing good parasiticidal properties [18].; however, a direct inhibitory effect on these enzymes either in the recombinant form or in *T. cruzi* exposed to the drugs was not evaluated. Other quinazoline-2,4 diamine derivatives have proved to inhibit recombinant DHFR from *Leishmania* [25]. 

The presence of nitro functional groups in compounds such as that present in Nfx and Bnz is common in several antiprotozoan and antitrypanosomatid drugs [26]; the presence of the nitro group in the **2**–**4** TAQ derivatives opens the possibility of a mode of action through their activation by parasite type I-nitroreductases, enzymes present in trypanosomatids but absent in the human host [27]. Since the nitro functional group may promote the production of reactive oxygen species depending on the chemical and on the dose [26], the involvement of the antioxidant trypanothione-based redox system of the parasite was analyzed for compounds **1**–**4**. No changes in T(SH)_2_ were observed at the ED_50_ that could explain the decrease in growth; this contrasts with other drugs such as buthionine sulfoximine, which clearly affects the thiol metabolites and enhances Bnz effect [28,29]. Notwithstanding, an effect on antioxidant defense at higher concentrations cannot be ruled out. 

## 4. Conclusions

A series of quinazoline-2,4,6-triamine derivatives were synthesized with moderate to good yields, up to 80%. The in silico analysis of ADME properties revealed that the compounds meet the criteria to be well absorbed following oral administration, with reduced probability of toxic effects. Compounds **2**–**4** characterized by nitro substituents in the benzoyl group bound to the ring of quinazoline showed high selectivity against the highly virulent *T. cruzi* Queretaro strain and showed low toxicity against a human model of fibroblasts. These characteristics encourage to continue its validation as potential use as antichagasic compounds. 

## 5. Methodology

### 5.1. Analytical Methods

The purity of the compounds was verified by melting point and mass spectrometry. Melting points were determined in open capillary tubes with an IA9000 Series Melting Point Apparatus (Bibby Scientific Limited, Staffordshire, UK) and they were uncorrected. The reactions were monitored by TLC on 0.2 mm precoated silica gel 60 F254 plates (E. Merck, Darmstadt, Germany) and visualized by irradiation with a UV lamp Spectroline Model ENF-240C (Spectro-UV, East Farmingdale, NY, USA). The mass spectrometry (JEOL JMS-800D Mass spectrometry instrument: JEOL, 11 Dearborn Road, Peabody, MA, USA) was performed by ESI-MS positive ion detection on a computer. The IR spectra were obtained on a spectrophotometer Pelkin ATX-Elmer (Perkins Engines Inc., 1600 W Kingsbury St Seguin, TX, USA). ^1^H NMR spectra and ^13^C NMR were acquired in DMSO_d6_ on a Varian 300 and 400 MHz equipment (Agilent Company, Santa Clara, CA, USA); chemical shifts were obtained in parts per million (ppm), by using the deuterated solvent itself as reference. Splitting patterns have been designated as follows: s, singlet; d, doublet; q, quartet; dd, doublet of doublet; t, triplet; m, multiplet; br s, broad singlet. Catalytic hydrogenations were carried out in a Parr Shaker Hydrogenation apparatus (Model Parr 3911,Parr Instrument Company, St, Moline, IL, USA). The starting materials 2-amine-5-nitrobenzonitrile, guanidine carbonate, benzoyl chloride, and aldehydes are commercially available (Sigma Aldrich, St. Louis, MI, USA). 

### 5.2. Preparation of 6-Nitroquinazoline-2,4-diamine

A mixture of 5.00 g (30.64 mmol) of 2-amino-5-nitro-benzonitrile, 4.17 g (23.03 mmol) guanidine carbonate and 2.05 g (36.77 mmol) of KOH in 25 mL of ethanol and 50 mL propanol was refluxed for 6 h. The warm suspension was separated by filtration and the solid collected was washed successively with water and methanol to obtain 5.35 g (yield: 85%) of an orange solid; m.p.: 359–362 °C; IR (νmax, cm^−1^): 3463, 3440, 3106 (NH), 1614, 1460 (C=C), 1661 (C=N), 1325, 1295 (C–NO_2_); ^1^H NMR (DMSO-d6, 400 MHz) δ ppm, 9.07 (1H, d, *J* = 2.50 Hz, Ar–H), 8.21 (1H, dd, *J* = 9.2 Hz, 2.50 Hz, Ar–H), 7.83 (2H, s, Ar–NH_2_), 7.21 (1H, d, *J* = 9.20 Hz, Ar–H), 6.76 (2H, s, Ar–NH_2_); ^13^C NMR (DMSO-d6, 101 MHz), δ ppm, 163.23, 162.97, 157.24, 139.13, 126.58, 124.85, 121.88, 108.82 (Ar–C); MS: *m*/*z* [M + H]^+^ 206.

### 5.3. Preparation of Quinazoline-2,4,6-triamine (TAQ)

A mixture of 3.00 g (17.12 mmol) of 6-nitroquinazoline-2,4-diamine, 0.33 g (10%) of Pd/C in 270 mL of methanol were placed in a Parr hydrogenation flask at a pressure of 60 lb/in^2^ for 1 h; the reaction end was consumed at 85 lb/in^2^. The suspension was then filtered to remove Pd/C catalyst and the filtrate was evaporated in vacuo to yield 0.24 g (yield: 94%) of a light yellow solid, m.p.: 250–252 °C; IR (νmax, cm^−1^): 3399, 3326, 3146 (NH), 1559 (C=N), 1520 (C=C); 1H NMR (DMSO- d6, 300 MHz), δ ppm, 7.01 (1H, d, *J* = 9.5Hz, Ar–H), 6.95–6.98 (1H, m, Ar–H), 6.94 (1H, s, Ar–H), 6.87 (2H, s, NH_2_), 5.48 (2H, s, NH_2_), 4.77 (2H, s, NH_2_); 13C NMR (DMSO-d6, 101 MHz), δ ppm, 104.00, 110.95, 123.52, 125.08, 142.14, 145.03, 158.31, 161.40 (Ar–C); MS: *m*/*z* [M + H]^+^ 176.

### 5.4. General Procedures for the Synthesis of ***1**–**4***

In this work, amine groups at 2- and 4-positions were not protected because the amine group, at 6-position, was the most reactive Treatment of TAQ with corresponding benzoyl chloride (d) yielded target compounds **1**–**4**.

*N*-(2,4-Diaminequinazolin-6-yl)benzamide (**1**)

Yield: 60%, m.p.: 302–303 °C; IR (ν_max_, cm^−1^): 3403, 3335 (N-H), 3110 (C-H aromatic), 1667 (C=O), 1641 (N-H amide),1625-1538 (C=C aromatic); ^1^H NMR (DMSO-d_6_, 400 MHz), δ ppm, 10.22 [(H_8_) (s, 1H)], 8.22 [(H_13_)(s, 1H)], 7.98 [(H_2_)(H_3_)(d, *J* = 7.02 Hz, 2H)], 7.51–7.65 [(H_4_, H_5_, H_6_, H_15_)(m, 4H)], 7.22[(H_14_) (d, *J* = 3.96 Hz, 1H)], 7.19 [(H_21_) (s, 2H)], 5.95 [(H_20_) (s, 2H)]. ^13^CNMR (DMSO-d_6_, 101 MHz), δ ppm, 162.47, 153.65, 145.75, 139.56, 131.12, 128.21, 127.70, 126.76, 124.15, 117.82, 110.12, 101.72 (Ar–C), 46.51 (Ar–CH_2_–NH); ESI-MS *m*/*z*: calcd for C_15_H_13_N_5_O [M + H]^+^ 279.303, found 279.140.

*N*-(2,4-Diaminequinazolin-6-yl)-2-nitrobenzamide (**2**)

Yield: 40%, m.p.: 264–265 °C; IR (ν_max_, cm^−1^): 3453, 3368 (N-H), 3060 (N-H amide), 1678 (C=C aromatic), 1514 (NO_2_), 1347 (NO_2_); ^1^H NMR (DMSO-d_6_, 400 MHz), δ ppm, 10.64 [(H_6_) (s, 1H)], 8.22 [(H_13_)(s, 1H)], 7.98 [(H_2_)(H_3_)(d, *J* = 7.02 Hz, 2H)], 7.51–7.65 [(H_4_, H_5_, H_6_, H_15_)(m, 4H)], 7.22[(H_14_) (d, *J* = 3.96 Hz, 1H)], 7.19 [(H_21_) (s, 2H)], 5.95 [(H_20_) (s, 2H)]. ESI-MS *m*/*z*: calcd for C_15_H_13_N_5_O [M + H]^+^ 324.303, found 324.140.

*N*-(2,4-Diaminequinazolin-6-yl)-3-nitrobenzamide (**3**)

Yield: 40%, mp: 289–291 °C; IR (ν_max_, cm^−1^): 3468, 3338 (N-H), 3089 (N-H amide), 1662 (C=C aromatic), 1525 (NO_2_), 1342(NO_2_); ^1^H NMR (DMSO-d_6_, 400 MHz), δ ppm, 10.61 [(H_6_) (s, 1H)], 8.84 [(H_10_)(s, 1H)], 8.22 [(H_3_), d, *J* = 2.2 Hz, 1H], 7.85 [(H_8_)(t, *J* = 8 Hz, 1H)], 7.79 [(H_7_)(H_9_)(m, 2H)], 7.66 [(H_2_), dd, *J* = 8.92 Hz, 1H], 7.22 [(H_1_), d, *J* = 8.92 Hz, H_1_], 7.17 [(H_4_)(s, 2H)], 7.66 [(H_4_, H_5_, H_6_, H_15_)(m, 4H)], 5.92 [(H_5_)(s, 2H)]. ESI-MS *m*/*z*: calcd for C_15_H_13_N_5_O [M + H]^+^ 324.303, found 324.142.

*N*-(2,4-Diaminequinazolin-6-yl)-4-nitrobenzamide (**4**)

Yield: 40%, mp: 320–323 °C; IR (ν_max_, cm^−1^): 3413, 3331 (N-H), 3106 (N-H amide), 1660 (C=C aromatic), 1512 (NO_2_), 1341(NO_2_); ^1^H NMR (DMSO-d_6_, 400 MHz), δ ppm, 10.57 [(H_6_) (s, 1H)], 8.84 [(H_10_)(s, 1H)], 8.22 [H_7_, s, 1H], 8.38 [(H_8_)(d, *J* = 8.8 Hz, 1H)], 7.79 [(H_7_)(H_9_)(m, 2H)], 7.65 [H_2_, dd, *J* = 8.92 Hz, 2.1 Hz, 1H], 7.22 [H_1_, d, *J* = 8.92 Hz, H_1_], 7.17 [(H_4_)(s, 2H)], 7.66 [(H_4_, H_5_, H_6_, H_15_)(m, 4H)], 7.22[(H_1_) (d, *J* = 3.96 Hz, 1H)], 7.19 [(H_21_) (s, 2H)], 5.95 [(H_20_) (s, 2H)], 5.92 [(H_5_)(s, 2H)]. ESI-MS *m*/*z*: calcd for C_15_H_13_N_5_O [M + H]^+^ 324.303, found 324.143.

### 5.5. General Procedures for the Synthesis of ***5**–**14***

The reaction of TAQ (0.43 mmol) was placed in 15 mL of HCl (6N) and stirred for 4 h to 90 °C. When reaction was completed, the mixture was cooled to 5 °C and neutralized by adding an appropriate amount of aqueous solution of NaOH (1 M). The precipitated solid formed was collected by filtration, washed with water, dried, and crystallized from a methanol/water mixture.

Compounds **5**–**10**, **12**, and **13** were previously reported by our research group [19] and in this work, the same procedures were repeated to obtain them.

*N*^6^-(2,5-Difluorobenzyl)quinazoline-2,4,6-triamine (**11**)

Yield: 70%, m.p.: 152–155 °C. IR: (*ν*_max_, cm^−1^): 3335, 3146 (NH), 1627 (C=C aromatic), 1488, 1183 (CF). ^1^H NMR (300 MHz, DMSO-d_6_, δ (ppm)), 7.72 (s, 2H, NH_2_), 7.25 (m, 2H, Ar-H), 7.06–7.17 (m, 4H, Ar-H), 6.41 (s, 2H, NH_2_), 6.23 (t, 1H, *J* = 6.2 Hz, NH), 4.37 (d, 2H, *J* = 5.9 Hz, CH_2_); ^13^C NMR (75 MHz, DMSO-d_6_, δ (ppm)), 161.96, 158.15, 156.59, 156.36, 143.60, 139.21, 128.91, 123.71, 122.09, 116.62, 115.66, 114.84, 110.47, 101.04, 54.11. Calcd for C_15_H_14_F_2_N_5_ [M + H]^+^ 302.1217, found 302.1212.

*N*^6^-(3,4,5-Trimethoxybenzyl)quinazoline-2,4,6-triamine (**14**)

Yield: 50%, m.p.: 206–208 °C. IR: (*ν*_max_, cm^−1^): 3342, 3141 (NH), 1646 (C=C aromatic), 1536, 1503 (C=N), 1125 (OCH_3_). Calcd for C_18_H_22_N_5_O_3_ [M + H]^+^ 356.1722, found 356.1717. ^1^H NMR (400 MHz, DMSO-d_6_, δ (ppm)), 8.51 (s, 2H, NH_2_), 7.65 (s, 2H, NH_2_), 7.24–7.39 (m, 1H, Ar-H), 7.22 (d, 1H, *J* = 8.3 Hz, Ar-H), 7.20 (s, 1H, Ar-H), 6.78 (s, 2H, NH_2_), 6.47 (t, 1H, *J* = 5.2 Hz, NH), 4.23 (d, 2H, *J* = 4.9 Hz, CH_2_), 3.75 (s, 6H, 2OCH_3_), 3.61 (s, 3H, OCH_3_); ^13^C NMR (101 MHz, DMSO-d_6_, δ (ppm)), 161.46, 1583.36, 152.75, 145.13, 142.75, 136.23, 135.72, 125.02, 123.52, 110.72, 105.25, 100.66, 59.95, 55.84, 47.49. 

### 5.6. Cell Culture

Human foreskin fibroblasts 1 (HFF1) were cultured in Petri dishes (60 mm) with Dulbecco’s Modified Eagle Medium (DMEM) from GIBCO (Grand Island, NY, USA), which contains 25 mM D-glucose, 4 mM L-glutamine, 0.03 mM phenol red, 5.3 mM KCl, and 110.3 mM NaCl and was supplemented with 10% fetal bovine serum (FBS) from GIBCO and an antibiotics mixture (100 µg streptomycin/mL and 100 U penicillin/mL (Sigma-Aldrich; Darmstadt, Germany). The cells were incubated at 37 °C under an atmosphere of 5% CO_2_ and 95% air. After reaching 80% confluence, the cells were collected by trypsinization and centrifugation, counted in a Neubauer chamber and used for drug treatment of for infection to obtain trypomastigotes. 

Epimastigotes of the *T. cruzi* Queretaro strain [22] were grown in LIT (liver infusion-tryptose) medium (0.5% liver infusion, 0.5% tryptose, 0.42% sodium phosphate, 0.4% NaCl, 0.2% glucose, 0.04% KCl), supplemented with 10% FBS (Biowest, Nuaillie, France), 0.025 mg/mL hemin, and antibiotics as above. Epimastigotes were grown at 28 °C. 

### 5.7. Trypomastigotes Obtention 

For the primary infection, 20,000 HFF1 cells/mL were inoculated in 5 mL DMEM supplemented with 10% FBS in a 175 cm^2^ flask (Corning; Corning, NY, USA;) and cultivated at 37 °C and 5% CO_2_ for 24 h for adherence. Afterwards, the medium was changed to DMEM supplemented with 2% FBS and the cells were infected with 10 × 10^6^ epimastigotes previously collected and washed with 10 mL DMEM without FBS. The cells were incubated for 48 h at 37 °C and 5% CO_2_ and later washed every 24 h with DMEM with 2%FBS until trypomastigotes liberation (5–7 days post infection). 

For the secondary infection, LLC-MK2 cells (epithelial cells from the kidney of an adult monkey) were infected in a similar fashion to the primary infection, but using 1 × 10^6^ trypomastigotes obtained from the primary infection. The released trypomastigotes were used immediately for drug treatment.

### 5.8. Drug Treatment

All the compounds were dissolved in DMSO as vehicle at a stock concentration of 10 mM.

Absorption spectra were obtained at wavelengths of 190–1100 nm, and the extinction coefficient were determined by the Lambert–Beer law. Those coefficients were used to calibrate the actual concentration of the compounds. Dilution of the compounds were prepared in DMSO.

Epimastigotes were grown in LIT medium at the exponential phase and aliquots of 1 × 10^5^ parasites were dispensed in 200 µL/well in a 96-well culture plate (Corning; NY, USA). Compounds were added at concentrations 0–150 µM in a maximum volume of 2 µL; care was taken that the DMSO vehicle did not surpass 1% concentration to prevent its toxicity. Control samples without the compounds were supplemented with the maximum amount of vehicle used. The plate was incubated at 28 °C for 24 h. Afterwards, the number of mobile epimastigotes (motility as parameter of viability) was counted in a Neubauer chamber. Relative growth is the difference of the final density of parasites after 24 h of treatment with the compound minus the density of parasites at time 0 of exposure; these differences were further normalized to the relative growth of the culture without compound. Plots of the percentage of relative growth against compound concentration were built and data adjusted to dose–response equation with the Origin Pro software (Origin Pro version 8.0) to calculate the concentration at which the variable is decreased by 50%. A similar protocol was performed, with a single initial pulse of the compound for the experiment after 5 days of treatment.

For trypomastigotes, 2 × 10^5^ trypomastigotes in 200 µL of DMEM-2%FBS were dispensed per well in a 96-well plate and they were exposed to the compounds of interest at concentrations of 0–150 µM and incubated at 37 °C for 3 h (longer times were not used due to their high transformation rate to amastigotes). The number of mobile trypomastigotes (motility as parameter of viability) was determined using a Neubauer chamber. Data were processed as above. 

To evaluate the effect on cell viability, HFF1 cells cultured and washed as above were dispensed in a 96 well plate to a concentration of 1 × 10^4^ cells/well in 0.2 mL and incubated for 24 h at 37 °C for adherence. Later, the medium was removed and compounds were added in the same culture medium at concentrations 0–150 µM and incubated for 24 h. To determine viability, cells were detached by trypsinization and then collected, and viability was determined by trypan blue exclusion. 

For all experiments, n means number of independent cultures (batches) tested.

### 5.9. Thiol Metabolites Determination

A culture of 200 mL at a density of 3 × 10^6^ epimastigotes/mL of LIT medium was separated into 7 aliquots of 25 mL each. The parasites were exposed to the TAQ derivatives **1**–**4** and canonical Bnz and Nfx to their different ED_50_ concentrations and were incubated for 24 h at 28 °C. Subsequently, 1 mL of each culture was separated for protein quantification by the Lowry method and the remaining culture was used to determine the contents of thiol metabolites by HPLC as described before [28, 29]. Briefly, both samples were centrifuged at 20,817× *g* for 10 min at 4 °C, the supernatant was removed and washed twice with phosphate buffered saline (PBS: 137 mM NaCl, 2.7 mM KCl, 10 mM Na_2_HPO_4_, 2 mM KH_2_PO_4_, pH = 7.4) and centrifuged under the same conditions. The pellet for protein quantification was resuspended in 100 μL of Lowry’s solution A (2% Na_2_CO_3_ in 0.1 M NaOH) and stored at −72 °C for further quantification. The pellet for thiol content was kept in liquid N_2_ and stored at −72 °C until quantification by HPLC.

The quantification of thiol metabolites was performed by high-performance liquid chromatography (HPLC) for which 90 μL of lysis buffer (20 mM HEPES buffer, pH = 7.4, 1 mM EDTA, 0.15 mM KCl) with 20 mM DTT was added to each sample and was subjected to three cycles of freezing in liquid N_2_ and thawing at 37 °C. The cell lysate was centrifuged at 20,817× *g* for 10 min and the supernatant was recovered and thiols were reduced with an excess of sodium borohydride (NaBH_4_) and incubated on ice for 10 min. Afterwards, 3% perchloric acid (HClO_4_) was added at a final concentration of 3% to stop the reaction. The sample was centrifuged at 20,817× *g* for 2 min, then the supernatant was filtered and 20 μL was injected into the Waters HPLC apparatus (Milford, MA, USA). The metabolites were separated using a reversed-phase column of 3 μm particle size and 30 m long (Altima, Columbia, MD, USA) and post-column derivatized with DTNB and absorbance determination with the Uv-Vis detector of the apparatus. Thiol standard curves were used for determination of the thiols in the problem samples. n means number of independent cultures (batches) tested.

## Figures and Tables

**Table 1:** In silico computational analyses of the **1**–**14** triamine quinazoline derivatives.

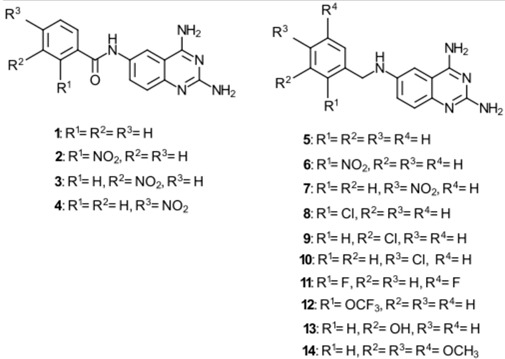									
No.	MW g/mol	*S* ^a^ mg/mL	ClogP ^b^ o/w	TPSA Å^2^	Lipinsky Rule Violations ^c^	Veber Rule Violations ^d^	%ABS ^e^	GI-ab ^f^	PAINS ^g^ Alert
**1**	279.30	0.22	1.64	106.92	0	0	72.11	High	0
**2**	324.29	0.30	0.98	152.74	0	1	56.30	Low	0
**3**	324.29	0.30	1.01	152.74	0	1	56.30	Low	0
**4**	324.29	0.30	1.03	152.74	0	1	56.30	Low	0
**5**	265.31	0.09	2.02	89.85	0	0	78.01	High	0
**6**	310.31	0.13	1.44	135.67	0	0	62.19	High	0
**7**	310.31	0.13	1.44	135.67	0	0	62.19	High	0
**8**	299.76	0.03	2.57	89.85	0	0	78.01	High	0
**9**	299.76	0.03	2.53	89.85	0	0	78.01	High	0
**10**	299.76	0.02	2.58	89.85	0	0	78.01	High	0
**11**	301.29	0.06	2.64	89.85	0	0	78.01	High	0
**12**	333.31	0.02	2.99	89.85	0	0	78.01	High	0
**13**	281.31	0.18	1.63	110.08	0	0	71.02	High	0
**14**	355.39	0.10	1.96	117.54	0	0	68.44	High	0

^a^ ESOL: Topological method; ^b^ Consensus; ^c^ Violations: MW > 500 Da, Log *p* > 5, hydrogen bond donors > 5, hydrogen bond acceptors > 10; ^d^ Violation (TPSA > 140 Å^2^) (TPSA: topological polar surface area), rotatable bonds > 10; ^e^ %ABS was obtained by following equation: %ABS =109 − (0.345 × TPSA); ^f^ GI-ab: gastrointestinal absorption of boiled egg white; ^g^ PAINS: Pan assay interference compounds.

**Table 2:** Anti-*T. cruzi* activity of the **1**–**14** triaminequinazoline derivatives.

		24 h			5 days			3 h
	Compound	Epi ED_50_	HFF1 LD_50_	*S.I.* *LD*_50_/*ED*_50_	Epi ED_50_	HFF1 LD_50_	*S.I.* *LD*_50_/*ED*_50_	Trypo LD_50_
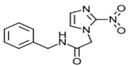	Bnz	9.4 ± 1.3 (3)	165 ± 27 (3)	*18*	17 ± 10 (3)	118 ± 14 (3)	*7*	183 (2)
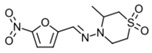	Nfx	3.8 ± 1.2 (3)	55 ± 6 (3)	*15*	11.8 ± 0.1 (3)	50 ± 7 (3)	*4*	86 (2)
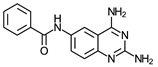	**1**	>100 (5)	>100 (6)	*1*	>100 (1)	>100 (2)	*1*	>150 (2)
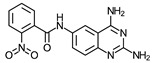	**2**	19 ± 2 (5)	>100 (6)	*≥5*	15 ± 3 (3)	>100 (2)	*≥6.7*	95 (2)
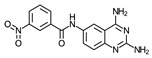	**3**	2.4 ± 0.4 (5)	>100 (6)	*≥42*	8.9 (2)	>100 (2)	*≥11*	50 (2)
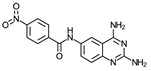	**4**	10 ± 0.4 (4)	>100 (6)	*≥10*	10 (2)	>100 (2)	*≥10*	60 (2)
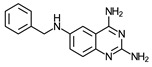	**5**	13 ± 3 (5)	>60 (4)	*≥5*	14 ± 2 (3)	26 (2)	*≥6*	>150 (2)
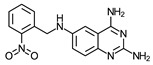	**6**	143 * (2)	–	*–*	–	–	*–*	–
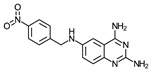	**7**	>100 * (2)	–	*–*	–	–	*–*	–
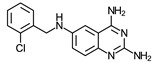	**8**	2.3 ± 0.5 (5)	8.6 ± 2 (4)	*4*	6.6 ± 0.5 (3)	8 (2)	*≥2*	59 (2)
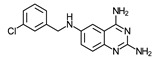	**9**	7.6 ± 2.5 (5)	85 ± 7 (6)	*11*	8.1 ± 1.8 (3)	>100 (2)	*≥12*	>150 (2)
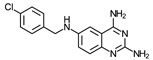	**10**	8.9 ± 2 (6)	116 ± 13 (6)	*13*	14 ± 1.2 (3)	>100 (2)	*≥7*	>150 (2)
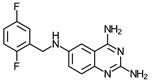	**11**	9 ± 1.4 (5)	>60 (4)	*≥7*	20 ± 2 (3)	>60 (1)	*≥3*	>150 (2)
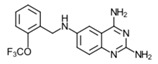	**12**	44 ± 3 (3) *	–	–	–	–	–	–
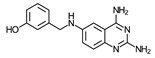	**13**	>100 * (2)	–	–	–	–	–	–
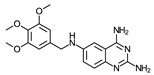	**14**	>100 * (2)	–	–	–	–	–	–

The inhibitory concentrations are expressed in µM. Lethal dose 50 (LD_50_) for HFF1 and trypomastigotes viability and effective dose (ED_50_) for epimastigote growth. S.I. = Selective index values calculated are the ratio LD_50_/ED_50_. “–” means not done. * initial screening done in 5 mL culture for 24 h. The values in parentheses are the number of independent cultures tested. Where indicated, the values are the mean ± standard deviation of at least three independent cultures or the mean when two independent cultures were evaluated.

## Data Availability

All data are available in the article.

## Supplementary Materials

The following supporting information can be downloaded at: https://www.mdpi.com/article/10.3390/molecules29184501/s1, Figure S1. Effect of TAQ-derivatives 6–7 and 12–14 on the growth of epimastigotes after 24 h treatment.

## Abbreviations

Bnz, benznidazole; CD, Chagas disease; Nfx, nifurtimox; TAQ, quinazoline-2,4,6-triamine.
