# Supplementary material for: Exploring Quinazoline Nitro-Derivatives as Potential Antichagasic Agents: Synthesis and In Vitro Evaluation
Source: Molecules. 2024 Sep 23;29(18):4501. doi: 10.3390/molecules29184501 (PMC11435156; doi:10.3390/molecules29184501)
Supplement: Supplementary file 1 [file molecules-29-04501-s001.zip › molecules-3183792-supplementary.pdf]

Supplementary Material for Manuscript

Exploring quinazoline nitro-derivatives as potential antichagasic agents: Synthesis and *in vitro* evaluation

Citlali Vázquez 1#, Audifás Salvador Matus-Meza 2#, Oswaldo Nuñez-Moreno 1, Brenda Michelle Barbosa-Sánchez 2, Victor Manuel Farías-Gutiérrez 2, Mariana Mendoza-Conde 1, Francisco Hernández-Luis 2* and Emma Saavedra1*

Supplementary Figure S1

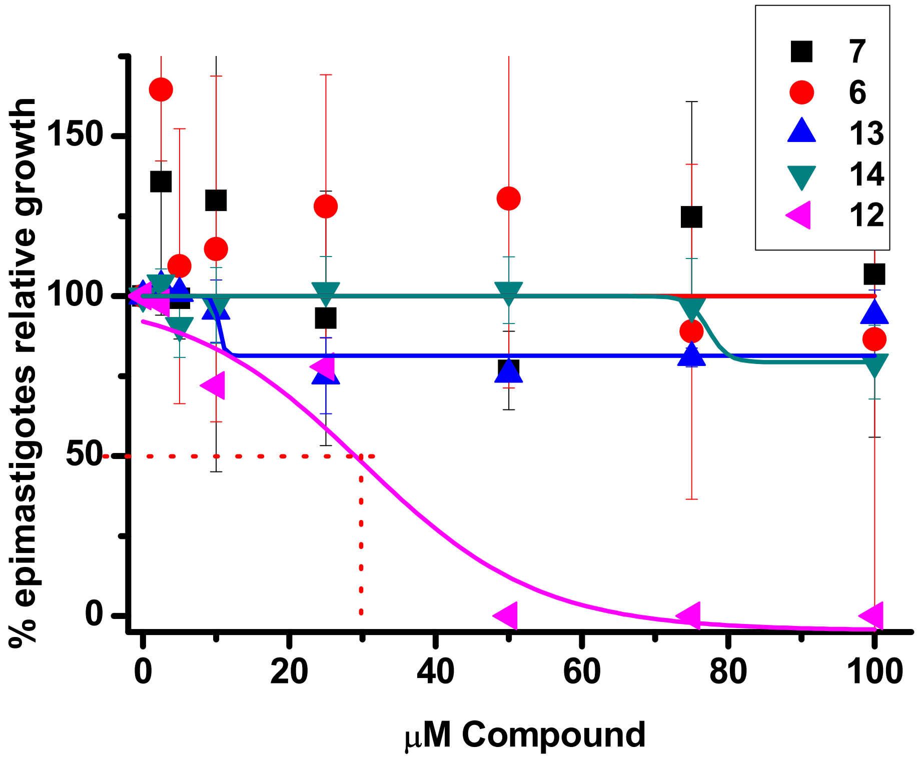

Fig. S1. Effect of TAQ-derivatives 6-7 and 12-14 on the growth of epimastigotes after 24 h treatment. Compound 12 had a high ED_{50} of 30 μM, so further investigation was discarded.
